# Body dissatisfaction and body mass in girls and boys transitioning from early to mid-adolescence: additional role of self-esteem and eating habits

**DOI:** 10.1186/1471-244X-12-35

**Published:** 2012-06-08

**Authors:** Mauno Mäkinen, Leena-Riitta Puukko-Viertomies, Nina Lindberg, Martti A Siimes, Veikko Aalberg

**Affiliations:** 1 Department of Psychiatry, Division of Adolescent Psychiatry, Helsinki University Central Hospital, University of Helsinki, Helsinki, Finland; 2 Department of Gynecology and Pediatrics, Helsinki University Central Hospital, University of Helsinki, Helsinki, Finland

## Abstract

**Background:**

In the transition from early to mid-adolescence, gender differences in pubertal development become significant. Body dissatisfaction is often associated with body mass, low self-esteem and abnormal eating habits. The majority of studies investigating body dissatisfaction and its associations have been conducted on female populations. However, some evidence suggests that males also suffer from these problems and that gender differences might already be observed in adolescence.

**Aims:**

To examine body dissatisfaction and its relationship with body mass, as well as self-esteem and eating habits, in girls and boys in transition from early to mid-adolescence.

**Methods:**

School nurses recorded the heights and weights of 659 girls and 711 boys with a mean age of 14.5 years. The Rosenberg Self-Esteem Scale and the Body Dissatisfaction subscale of the Eating Disorder Inventory were used as self-appraisal scales. Eating data were self-reported.

**Results:**

The girls were less satisfied with their bodies than boys were with theirs (mean score (SD): 30.6 (SD 12.2) vs. 18.9 (SD 9.5); p < 0.001). The girls expressed most satisfaction with their bodies when they were underweight, more dissatisfaction when they were of normal weight and most dissatisfaction when they had excess body weight. The boys also expressed most satisfaction when they were underweight and most dissatisfaction when they had excess body weight. The boys reported higher levels of self-esteem than did the girls (mean (SD): 31.3 (4.8) vs. 28.0 (5.9); p < 0.001). The adolescents self-reporting abnormal eating habits were less satisfied with their bodies than those describing normal eating habits (mean (SD): 33.0 (12.9) vs. 21.2 (10.2); p < 0.001).

**Conclusions:**

Body mass, self-esteem and eating habits revealed a significant relationship with body dissatisfaction in the transitional phase from early to mid-adolescence in girls and boys, but significant gender differences were also found.

## Background

Adolescence is a period of life when individuals transfer from childhood and their biological, cognitive, psychological and social characteristics rapidly change as they become more adult-like. This challenging developmental stage is initiated by pubertal onset and can be divided into three periods: early (ages 12 to 14 years), middle (ages 15 to 16 years) and late adolescence (ages 17 to 19 years and beyond) [1]. In the transition from early to mid-adolescence, gender differences in pubertal maturation are significant. Girls are already post-pubertal, having reached their final height as well as accumulated adipose tissue at specific sites. Boys, however, represent the full scale of pubertal development from early to post-puberty, most of them having their lifetime lowest body fat content due to the simultaneous growth spurt in height [2].

Body dissatisfaction, the subjective evaluation of one’s figure or body part, has been conceptualized to be an important part of body image disturbance [3,4]. In three recent large community-based studies, the proportion of adolescent girls reporting body dissatisfaction varied between 24 % and 46 %, whereas the respective proportions of boys ranged from 12 % to 26 % [4-6]. Body dissatisfaction appears to either remain stable or increase during adolescence among girls [7,8]. It has been speculated that puberty precipitates body dissatisfaction in girls, who accrete more adipose tissue, which in turn moves them away from the current thin beauty ideal [4,9]. Among boys, body dissatisfaction has been reported to either decrease or remain stable as they move towards adulthood [7,8]. However, boys are nowadays known to be under increasing pressure to meet their unrealistic lean and muscular body ideal [10-12].

Body mass is the most consistent biological factor correlated with body dissatisfaction, although the relation seems to differ between genders [4,12,13]. Boys have been reported to feel dissatisfaction with their bodies when either below or above average weight, and to be most satisfied when they are of average weight [4,10,14]. In contrast, girls showed a positive linear relationship, such that their body dissatisfaction increased as a function of body weight [4,14]. Dissatisfaction with one’s body tends to manifest in attempts at weight loss in girls, whereas dissatisfaction in boys can either appear as weight gain or weight loss [15].

Self-esteem can be described as a favourable or unfavourable attitude towards oneself [16]. Low self-esteem is a lack of respect for oneself, with feelings of unworthiness, inadequacies and deficiencies [16]. Self-esteem is more strongly associated with age among boys than girls, and self-esteem has been reported to be higher among boys than girls during adolescence [17,18]. The extent of being overweight has been described to be inversely correlated with self-esteem, although the magnitude of the relationship has been found to be only modest, with self-esteem scores of overweight adolescents within the normal range [19,20]. Apart from perceived overweight, body dissatisfaction is also often associated with low self-esteem, especially in girls [21-23]. In fact, body dissatisfaction has been reported to be a risk factor for low self-esteem in girls in early adolescence and in boys in mid-adolescence [24].

Abnormal eating habits may be associated with body dissatisfaction [25]. Dissatisfaction is thought to increase the risk of eating pathology through two central mechanisms [26]. The first suggested pathway is that body dissatisfaction leads to increased dietary restraint, which subsequently leads to an increased likelihood of anorexic and bulimic behaviour**.** The another suggested pathway is that body dissatisfaction leads to increases in negative emotional feelings, which in turn increase the risk of binge eating, as some individuals overeat to ameliorate such adverse emotions. However, it has been reported that men who are dissatisfied with their bodies appear to be less likely to diet or attempt to lose weight than women [27].

The aim of the present study was to examine body dissatisfaction and its relationship with body mass, as well as self-esteem and eating habits, in girls and boys in transition from early to mid-adolescence. The differences in pubertal maturation in this age group indicate that gender differences might exist. Our hypotheses were that: 1. girls would be more dissatisfied with their bodies than boys, and the relationship between body dissatisfaction and body mass would differ between genders; 2. body dissatisfaction would be related to self-esteem and the relationship would be gender-specific; and 3. eating habits would be associated with body dissatisfaction, especially in girls.

## Material and Methods

### Participants

This cross-sectional study was performed on adolescent girls and boys attending the 8th grade at 24 secondary schools in the city of Helsinki, Finland, in 2003 and 2004. The mean age of the participants was 14.5 (SD 0.3) years. Although the general population is relatively homogeneous in Helsinki, the schools were selected in order to cover all the representative socio-economic groups across the city districts. The sample included state, municipal and private schools. All participants attended ordinary education programmes and they spoke Finnish as their mother tongue. Of the 2 286 students, 1 370 participated in the study (659 girls and 711 boys). The overall participation rate was 61.4 % for girls and 58.6 % for boys. Single missing values were imputed. However, in 27 (2.0 %) cases for which there were systematic missing values, their data were excluded from the study. Accordingly, the final sample consisted of 650 girls and 693 boys**.**

### Procedure

Teachers of the selected schools received written information about the study and were present during the 60-minute data collection visit when the participants completed the questionnaires. The questionnaire covered details of the participants’ health- and food-related habits in addition to recording the measurements described below. The questionnaire also contained numerical codes to match and identify the participants.

### Measures

#### *Body dissatisfaction*

The Body Dissatisfaction subscale of the Eating Disorder Inventory (EDI) [3,28] was used to measure body dissatisfaction. The subscale consists of 9 items that are all rated on a 6-point Likert-scale, with response options ranging from “always” to “never”. Higher scores on the scale indicate a greater dissatisfaction with one’s body. The EDI was initially developed for women and is well validated in female populations [3,28-30]. However, its validity with respect to studying body dissatisfaction in adolescent boys has only recently been reported [31]. In the present study, the internal consistency of the EDI was found to be acceptable for both sexes (Cronbach’s alpha 0.94 for girls and 0.89 for boys).

#### *Body mass*

School nurses measured the body weights and heights of the participants. The body mass index (BMI: the body weight in kilograms divided by the square of the height in metres (kg/m^2^)) was used to reflect the degree of excess body weight. Earlier studies have indicated that the reference values increase with age and BMI may be a valid measure of adipose cover among adolescents [32]. Consequently, the respective cut-off points of 25 and 30 kg/m^2^ for overweight and obesity commonly used for adults were substituted with the international lower cut-off points of BMI percentiles for adolescents as described by Cole et al. [33] (Table 1). In addition, the <5th percentile of the reference curves for Finnish children was used as a cut-off point for being underweight in the present study [34].

**Table 1:** BMI cut-off points and weight status among 1343 adolescent girls and boys

***Weight status***	***BMI cut-off points***		***Frequencies***			
	**Girls**	**Boys**	**Girls ** **N**	**(%)**	**Boys ** **N**	(%)
**Underweight**	< 16.40*	< 16.00*	28	(4.3)	25	(3.6)
**Normal weight**	16.40-23.59	16.00-22.95	550	(84.6)	567	(81.8)
**Overweight**	> 23.60**	> 22.96**	63	(9.7)	78	(11.3)
**Obese**	> 28.81**	> 27.98**	9	(1.4)	23	(3.3)
**Total**			650	(100.0)	693	(100.0)

*The cut-off points of BMI for being underweight at the age of 14.5 years are based on growth data for Finnish children.

**The international cut-off points of BMI for being overweight and being obese at the age of 14.5 years in adolescent girls and boys.

#### *Self-esteem*

Self-esteem was measured using the Rosenberg Self-Esteem Scale [16]. The scale comprised 10 self-appraisal statements, each rated as positive or negative. Each statement has four separate response options (from 1 = “not at all true of me” to 4 = “very true of me”), and higher scores reflect a greater level of self-esteem. The Rosenberg Self-Esteem Scale has been widely used in measuring self-esteem among adolescents [16,24,35,36]. Its reliability and validity are well documented [37]. In the present study, internal consistency was acceptable (Cronbach’s alpha 0.86 for girls and 0.79 for boys).

#### *Eating habits*

Eating habits were assessed from answers to the following question: “Which of following best describes you?” with four options: “It’s easy for me to eat approximately the amount I need” (normal eating); “I quite often eat more than I actually need “(overeating); “I often try to restrict my eating” (restrictive eating); and “Occasionally, I’m on a strict diet or I overeat” (alternating restrictive eating/overeating. The last three response options were considered as being indicative of abnormal eating habits.

### Statistical methods

The analyses were performed on girls and boys as separate groups. Missing value analysis and imputation were carried out using the expectation maximization method. An independent-samples t-test and one-way ANOVA were used to compare the means. The chi-squared (χ^2^) test was used to compare frequencies**.** The differences in the correlations were estimated using an interaction term between gender and the explanatory variable. Eating habits were dichotomized (normal eating/abnormal eating) for regression analysis. Cohen’s d effect sizes obtained from the t-tests were calculated to describe practical significance values. The Cohen effect sizes classes of 0.2 as small, 0.5 medium and 0.8 large were used [38]. Linear regression analysis was performed to measure the associations between body dissatisfaction and other variables, despite the fact that some distributions were skewed and some relationships departed from a linear relationship. Neither stepwise nor hierarchical models were used. The distribution free method CATREG produced very similar results to linear regression analysis [39]. Linear regression analysis was also used when 5 % of the lowest and highest scores were eliminated, but the elimination did not influence the results. BMI squared and LOWESS (locally weighted scatterplot smoothing) regression were compared and used to describe the nonlinearity between variables [40]. The data were analyzed using SPSS for Windows, version 18.0 [41]. P-values < 0.05 indicated statistical significance in all tests.

### Ethics

The Ethics Committee of the Hospital for Children and Adolescents at Helsinki University Central Hospital, Helsinki, Finland approved the study. Letters outlining the nature of the study were sent to the parents or guardians of the under-aged participants. Either the active or passive consent of parents or guardians was obtained. The participants were also requested to provide their own written permission when completing the questionnaires in the study session.

## Results

We observed that the girls experienced more body dissatisfaction than the boys (Table 2). Although body dissatisfaction scores were gender specific, the individual scores ranged from a minimum of 9 to a maximum of 54 among both sexes. Even so, as many as 24 out of 650 girls (3.7 %) but only one out of 693 boys (0.1 %) provided maximal body dissatisfaction scores (p < 0.001). The average BMI value was similar in the girls and boys, being 20.3 kg/m^2^ (Table 2). The prevalence of adolescents with excess body weight was 11.1 % for the girls and 14.6 % for the boys (p = 0.056). Moreover, 1.4 % of the girls and 3.3 % of the boys met the criteria for obesity (p = 0.020) (Table 1)..

**Table 2:** Gender-specific differences in body dissatisfaction and self-esteem scores and body mass index among 1343 adolescent girls and boys

	***Girls (N = 650)***		***Boys (N = 693)***			
	**Mean**	**(SD)**	**Mean**	**(SD)**	**p**	**Cohen’s d**
**BD of EDI**	30.6	(12.2)	18.9	(9.5)	< 0.001	1.1^*^
**BMI (kg/m**^**2**^**)**	20.3	(2.9)	20.3	(3.2)	> 0.05	0.0
**RSES**	28.0	(5.9)	31.3	(4.8)	< 0.001	0.6^**^

BD of EDI - Body Dissatisfaction subscale of the Eating Disorder Inventory, BMI - body mass index, RSES - Rosenberg Self-Esteem Scale.

^*^Cohen’s d = large.

^**^Cohen’s d = medium

**Figure 1:**
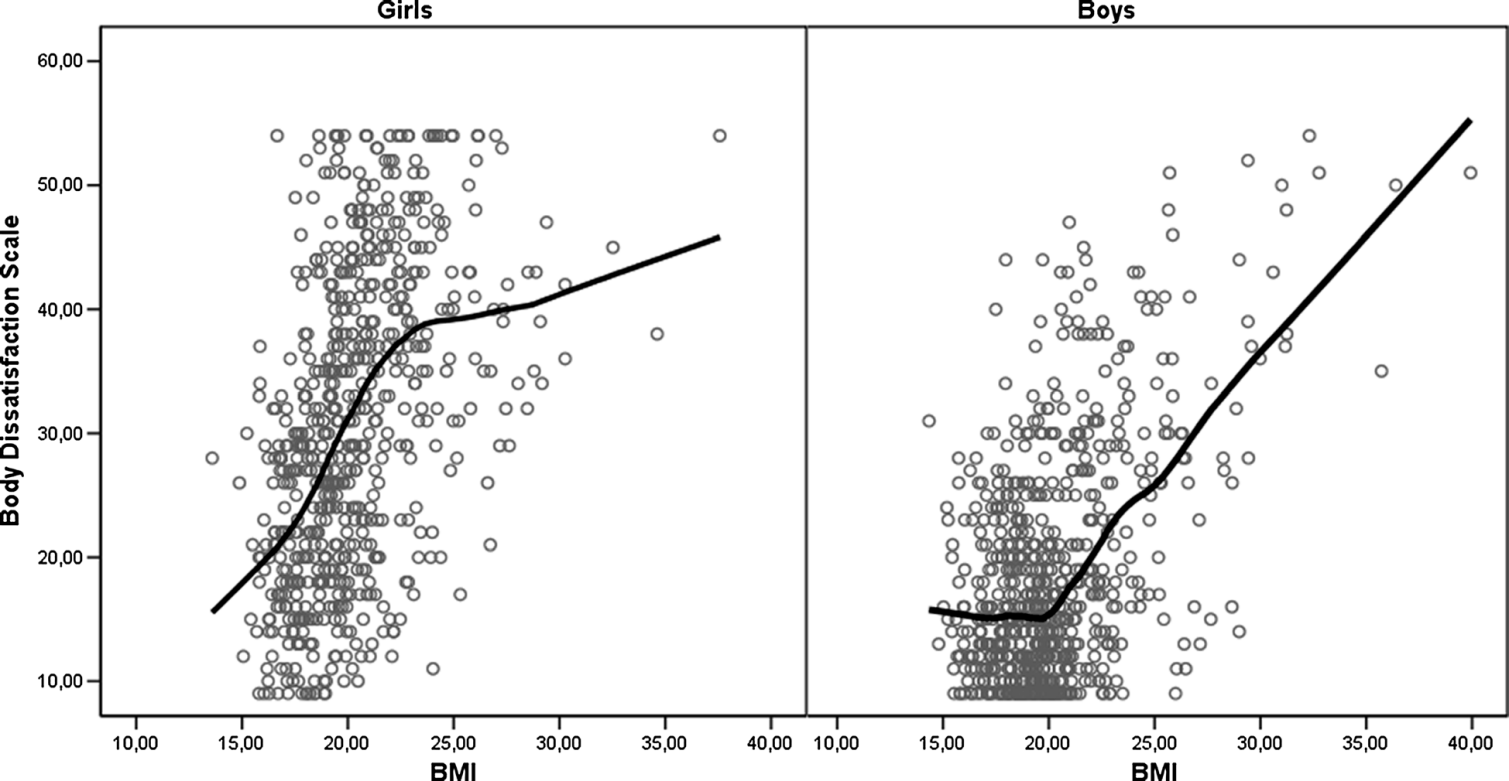
**Associations of the Body Dissatisfaction Scale with BMI in girls and boys described by a LOWESS curve.** Body dissatisfaction scores ranged from 9 to 54.

The girls expressed most satisfaction with their bodies when they were underweight, more dissatisfaction when they were of normal weight and most dissatisfaction when they had excess body weight (body dissatisfaction: mean score (SD) = 20.1 (8.1) vs. 30.0 (11.2) vs. 39.4 (10.3); p < 0.001). The boys expressed also most satisfaction when they were underweight and most dissatisfaction when they had excess body weight (body dissatisfaction: mean score (SD) = 16.6 (6.2) vs. 17.3 (8.0) vs. 28.8 (SD 11.6); p < 0.001) (Figure 1). We found a similar association between body dissatisfaction and BMI for the girls and boys (p < 0.001) (Table 3). On closer examination, differences between sexes emerged for body dissatisfaction and weight status (Figure 1). Among the girls, we found an association between body dissatisfaction and BMI among those of normal weight (r = 0.43, p < 0.001), but not among those who were underweight (r = −0.28, p **=** 0.16, n.s.) or had excess body weight (r = 0.10, p **=** 0.43, n.s). Among the boys, an association between body dissatisfaction and BMI was found among those of normal weight (r = 0.23, p < 0.001) and also among those with excess body weight (r = 0.50, p < 0.001). However, no such relationship between body dissatisfaction and BMI was found in underweight boys (r = −0.29, p = 0.16, n.s).

**Table 3:** Linear regression analysis, coefficients, with the Body Dissatisfaction subscale of the Eating Disorder Inventory as the dependent variable among 1343 adolescent girls and boys

		***Unstandardized Coefficients***		***Standardized Coefficients***			***Correlations (zero-order)***	***Unique proportion of the variance***
		**B**	**Std. Error**	**Beta**	**t**	**p**	**Pearson**	**%**
**Girls **(N = 650)****	BMI	1.76	0.15	0.42	12.05	< 0.001	0.46	11.4
	BMI squared	−0.71	0.18	−0.13	−3.91	< 0.001	0.13	1.2
	RSES	−0.80	0.06	−0.39	−13.04	< 0.001	−0.52	13.4
	EH	6.04	0.79	0.24	7.67	< 0.001	0.48	4.6
**Boys **(N = 693)****	BMI	1.03	0.11	0.35	9.03	< 0.001	0.50	6.9
	BMI squared	0.51	0.14	0.14	3.67	< 0.001	−0.41	1.1
	RSES	−0.68	0.06	−0.34	−11.46	< 0.001	−0.39	11.4
	EH	3.49	0.70	0.15	4.97	< 0.001	0.35	2.1

BMI - body mass index, RSES - Rosenberg Self-Esteem Scale, EH - eating habits, Squared multiple correlation x 100 (girls) = 48.9 %, Squared multiple correlation x 100 (boys) = 41.6 %.

**Figure 2:**
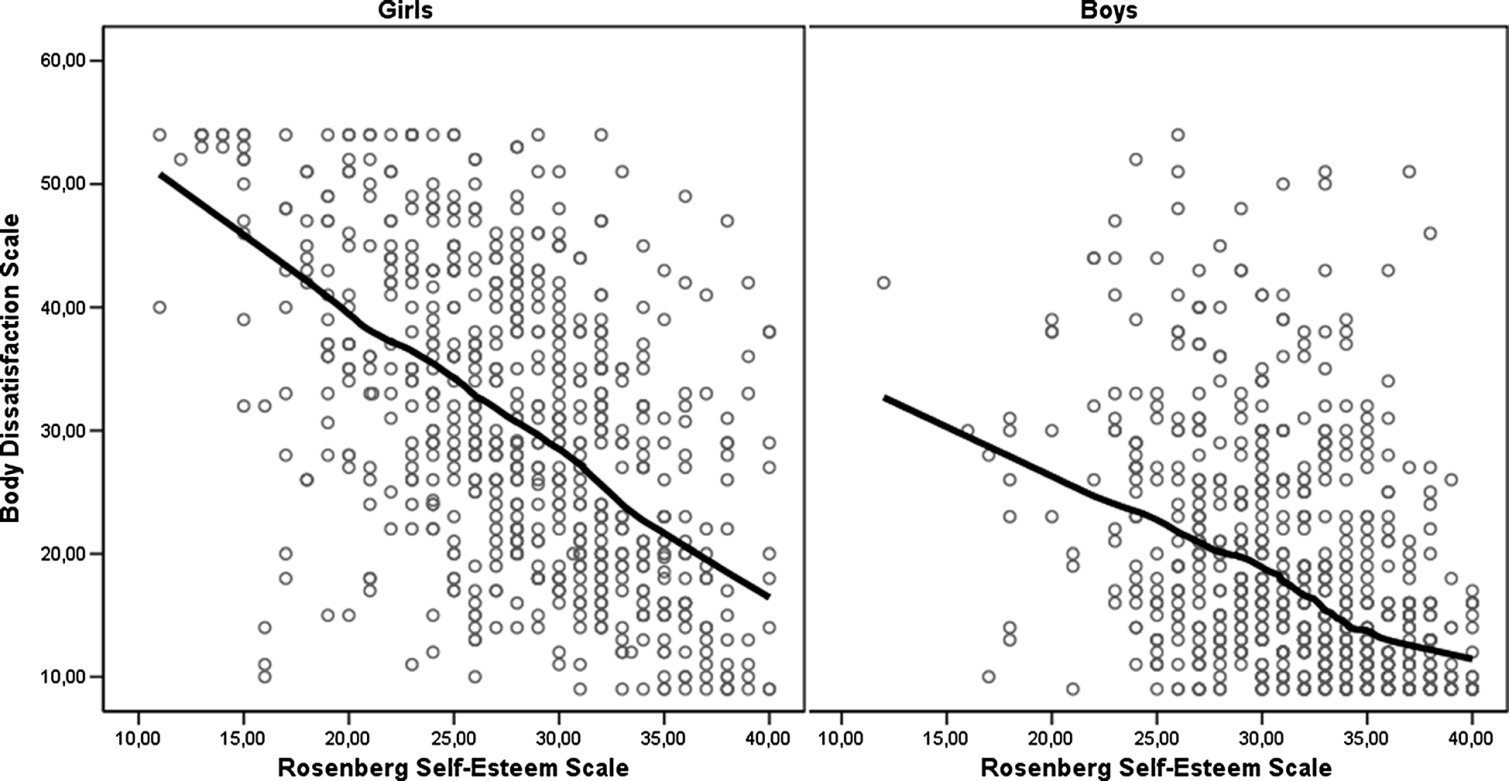
**Associations of the Body Dissatisfaction Scale with the Rosenberg Self-Esteem Scale in girls and boys described by a LOWESS curve.** Body dissatisfaction scores ranged from 9 to 54.

Self-esteem scores were higher for the boys than girls (Table 2). We observed a linear association between body dissatisfaction and self-esteem among girls and boys, although the correlations differed between genders (p = 0.005) (Figure 2; Table 3).

Eating habits differed between the girls and boys (Table 4). A normal eating pattern was more prevalent among the boys than the girls (p *<* 0.001). Subjectively expressed overeating (p *=* 0.026), restrictive eating (p < 0.001) and alternating restrictive eating/overeating (p *=* 0.010) were more prevalent among the girls than among the boys (Table 4). The relationship between eating habits and body dissatisfaction was stronger among girls than boys (p < 0.001) (Table 3). One-way ANOVA demonstrated that differences between the means of the body dissatisfaction scores and BMI values according to the four eating habits were significant in both genders (p < 0.001). The girls and boys who reported abnormal eating had higher BMI values than adolescents self-reporting normal eating (mean (SD): 21.6 kg/m^2^ (3.6) vs. 19.7 kg/m^2^ (2.6); p < 0.001). They also had higher body dissatisfaction scores (mean (SD): 33.0 (12.9) vs. 21.2 (10.2); p < 0.001). BMI, BMI squared, self-esteem and eating habits combined explained 48.9 % of the total variation in body dissatisfaction in girls and 41.6 % in boys (Table 3**).** Respectively, regression analysis using the LOWESS variable instead of BMI squared explained significantly more variation: 49.8 % in girls and 42.6 % in boys (p < 0.001). Thus the LOWESS variable better described the nonlinearity between body dissatisfaction and BMI than the quadratic component, although in regression analysis it was also significant (p < 0.001). The relationship consisted of two different linear components around a turning point in both genders rather than a pure quadratic relationship (Figure 1)..

**Table 4:** Self-reported eating habits among 1343 adolescent girls and boys

	**Girls N**	**(%)**	**Boys N**	**(%)**
**NE**	418	(64.3)	540	(77.9)
**OE**	120	(18.5)	97	(14.0)
**RE**	82	(12.6)	45	(6.5)
**RE/OE**	30	(4.6)	11	(1.6)
**Total**	650	(100.0)	693	(100.0)

NE - normal eating, OE - overeating, RE - restrictive eating, RE/OE - alternating restrictive eating/overeating, χ^2^ = 36.22, df = 3, p < 0.001

## Discussion

As hypothesized and consistently with previous studies, the girls were less satisfied with their bodies than boys were with theirs [4,7,8]. On closer examination, the girls expressed the greatest satisfaction with their bodies when they were underweight. The girls who were overweight or of normal weight expressed lower body satisfaction. This finding is in line with previous research documenting the impact of a greater body mass on body dissatisfaction in adolescent girls [6,8,24]. Interestingly, a recent study indicated that body satisfaction was protective against increased body mass, even among girls who were overweight. Findings such as this point to the importance of helping adolescent girls, regardless of their size, to develop a positive sense of their bodies [42].

In some previous studies among adolescent boys, the relationship between body dissatisfaction and body mass has been described to be quadratic, which indicates that boys express most dissatisfaction with their bodies when they are either below or above average weight and most satisfaction when they are of average weight [4,14]. We also found a significant quadratic component, but LOWESS regression described the nonlinearity better. In fact, the curve had an inverse L-shape, indicating that body dissatisfaction was related to being overweight or obese, but not to being underweight. Age does not explain the difference, since a quadratic component has been described in both younger and older boys than those of our sample [4,14]. However, cultural norms might give an explanation for this difference: a muscular male body ideal may not be strong enough to cause body dissatisfaction in Finnish underweight boys in transition from early to mid-adolescence. This hypothesis should, however, be tested in future studies.

As hypothesized, self-esteem and body dissatisfaction were negatively correlated in both genders, but the correlation was stronger among the girls. From the perspective of clinical work, the findings highlight the importance of strengthening the self-esteem of adolescents expressing abnormal eating, which in turn may reduce their body dissatisfaction and consequently lower the risk of developing clinical eating disorders [43].

A normal eating pattern was significantly more prevalent among the boys than the girls in our study, which is in line with the fact that both clinical eating disorders and subclinical eating pathology are typically problems of young females [44,45]. The relationship between eating habits and body dissatisfaction was found in both genders, but, as hypothesized, it was stronger among the girls. In line with two previous studies, the adolescents self-reporting abnormal eating habits were less satisfied with their bodies than those describing normal eating habits [25,26]. We found that the girls and boys reporting abnormal eating showed higher BMI values than adolescents self-reporting normal eating. According to a previous follow-up study among adolescents, dieting and unhealthy weight control behaviours predicted greater body mass increases in females and males, as compared with cases with no such behaviour. Associations were found in both overweight and non-overweight participants [46]. Research is needed to assess whether helping adolescents substitute dieting and unhealthy weight control behaviours with healthier behavioural patterns results in long-term improvements in weight status [46].

The prevalence of overweight and obesity in adolescence has been reported to vary between 10 % and 20 % in most European countries, and consistently with this was approximately 11 % among the girls and 15 % among the boys in the present study [47,48]. Our results were also in accordance with the findings of a study on a nationally representative sample of Finnish 14-year-old girls and boys [49]. In both the national study and the present investigation, overweight and obesity appeared to be more prevalent among boys than girls.

The strength of the present study was its scale covering 24 secondary schools in the city of Helsinki*.* The overall participation rate of the present study was approximately 60 %. A traditional nationwide school survey carried out biannually in Finnish comprehensive schools (grades 8th and 9th) with the same data collection method has repeatedly reported a participation rate of approximately 80 % [50]. Consequently, the participation rate of the present study cannot be regarded as excellent or good, but we consider it acceptable. Unfortunately, we were unable to specify any attributes of the dropout group. Nonetheless, it is possible that the adolescents with the most marked eating problems might have refused to participate in the study because the study methodology included weight measurements performed by school nurses. The BMI values used in the present study were calculated from measurements taken by professional school nurses rather than self-reported values, as self-reported data are known to underestimate the prevalence of being overweight [51].

The body dissatisfaction subscale of the EDI was used to measure body dissatisfaction in this study. It is a widely used questionnaire for both girls and boys. However, it does not contain questions about the desire to be bigger, which is a factor relevant to some adolescent boys [12,15]. For this reason, it has been speculated that the EDI is perhaps not an ideal measure to examine the correlates of the desire to gain weight [15]. Nevertheless, there is evidence of its usefulness in scoring body dissatisfaction in boys [31,52,53]. One must bear in mind that the assessment of the eating habits was self-reported and limited to one question. Consequently, such data do not necessarily reflect actual eating behaviours but subjective ideas or memories of eating habits. However, Keski-Rahkonen et al. have previously used the same question with same response options in their large study on Finnish twins [54]. Also, the number of underweight boys was small and the results must be interpreted with caution.

## Conclusions

Body mass, self-esteem and eating habits revealed a significant relationship with body dissatisfaction in the transitional phase from early to mid-adolescence in girls and boys, but significant gender differences were also found.

## Competing interests

The authors report no competing interests.

## Authors’ contributions

MM, L-RP-V, MAS, and VA participated in the design of the study. MM, NL, and MAS carried out the drafting of the manuscript. MM, L-RP-V, NL, MAS, and VA carried out critical revision of the manuscript. MM, NL, MAS, and VA obtained funding for the study. All authors read and approved the final manuscript.

## Pre-publication history

The pre-publication history for this paper can be accessed here:

http://www.biomedcentral.com/1471-244X/12/35/prepub
